# Polyclonal HER2-specific antibodies induced by vaccination mediate receptor internalization and degradation in tumor cells

**DOI:** 10.1186/bcr3204

**Published:** 2012-06-07

**Authors:** Xiu-Rong Ren, Junping Wei, Gangjun Lei, Jiangbo Wang, Jiuyi Lu, Wenle Xia, Neil Spector, Larry S Barak, Timothy M Clay, Takuya Osada, Erika Hamilton, Kimberly Blackwell, Amy C Hobeika, Michael A Morse, H Kim Lyerly, Wei Chen

**Affiliations:** 1 Department of Medicine, Duke University Medical Center, 595 Lasalle Street, Durham, NC 27710, USA; 2 Duke Comprehensive Cancer Center, Duke University Medical Center, 203 Research Drive, Durham, NC 27710, USA; 3 Division of Medical Oncology, Department of Medicine, Duke University Medical Center, 203 Research Drive, Durham, NC 27710, USA; 4 Department of Cell Biology, Duke University Medical Center, 171 Research Drive, Durham, NC 27710, USA; 5 GlaxoSmithKline Biologicals S.A, 1330 Rixensart, Belgium; 6 Department of Medicine, Oncology, Duke University Medical Center, 40 Duke Medicine Circle, Durham, NC 27710, USA; 7 Division of General Surgery, Department of Surgery, Duke Comprehensive Cancer Center, Duke University Medical Center, 203 Research Drive, Durham, NC 27710, USA

## Abstract

**Introduction:**

Sustained HER2 signaling at the cell surface is an oncogenic mechanism in a significant proportion of breast cancers. While clinically effective therapies targeting HER2 such as mAbs and tyrosine kinase inhibitors exist, tumors overexpressing HER2 eventually progress despite treatment. Thus, abrogation of persistent HER2 expression at the plasma membrane to synergize with current approaches may represent a novel therapeutic strategy.

**Methods:**

We generated polyclonal anti-HER2 antibodies (HER2-VIA) by vaccinating mice with an adenovirus expressing human HER2, and assessed their signaling effects *in vitro *and anti-tumor effects in a xenograft model. In addition, we studied the signaling effects of human HER2-specific antibodies induced by vaccinating breast cancer patients with a HER2 protein vaccine.

**Results:**

HER2-VIA bound HER2 at the plasma membrane, initially activating the downstream kinases extracellular signal-regulated protein kinase 1/2 and Akt, but subsequently inducing receptor internalization in clathrin-coated pits in a HER2 kinase-independent manner, followed by ubiquitination and degradation of HER2 into a 130 kDa fragment phosphorylated at tyrosine residues 1,221/1,222 and 1,248. Following vaccination of breast cancer patients with the HER2 protein vaccine, HER2-specific antibodies were detectable and these antibodies bound to cell surface-expressed HER2 and inhibited HER2 signaling through blocking tyrosine 877 phosphorylation of HER2. In contrast to the murine antibodies, human anti-HER2 antibodies induced by protein vaccination did not mediate receptor internalization and degradation.

**Conclusion:**

These data provide new insight into HER2 trafficking at the plasma membrane and the changes induced by polyclonal HER2-specific antibodies. The reduction of HER2 membrane expression and HER2 signaling by polyclonal antibodies induced by adenoviral HER2 vaccines supports human clinical trials with this strategy for those breast cancer patients with HER2 therapy-resistant disease.

## Introduction

Breast cancers overexpressing HER2 have an aggressive clinical course. Despite the proven effectiveness of the HER2-specific mAb trastuzumab (Herceptin) and the dual epidermal growth factor receptor (EGFR) and HER2 receptor tyrosine kinase inhibitor lapatinib (Tykerb), disease progression and the rate of cancer-related deaths remain unacceptably high. HER2 remains overexpressed on cells that develop resistance to either anti-HER2 mAbs or tyrosine kinase inhibitor, which may be partly responsible for these failures in therapy, because additional blockade by combining trastuzumab and lapatinib provides clinical benefit [1,2]. Recent preclinical and clinical studies using novel mAbs that prevent HER2 and HER3 dimerization also appear to be effective, suggesting that persistent HER2 signaling is a cause of treatment failure [3,4]. The depletion of HER2 from the surface of resistant tumors cells by novel agents may therefore provide a means for reducing tumor aggressiveness and improving patient survival.

HER2/neu together with HER1 (EGFR), HER3, and HER4 comprise the EGFR family of plasma membrane tyrosine kinases [5,6]. HER2, in contrast to the other three receptors, is an orphan with no recognized endogenous ligand; nevertheless, plasma membrane-localized HER2 signals as a consequence of intrinsic tyrosine kinase activity. The HER2 receptor forms homodimers as well as heterodimer pairs with HER1, HER3, or HER4 [7-9]. HER2/3 heterodimers are the most potent activators of the phosphatidylinositol-3-kinase (PI3K)-Akt cell survival pathway. The binding of the HER1 ligand epidermal growth factor (EGF) and the HER3 ligand neuregulin to extracellular domains of HER1 and HER3, respectively, leads to receptor activation and the formation of homodimers or heterodimers. The increased kinase activity of heterodimeric partners leads to the transactivation of HER2 and phosphorylation of tyrosine residues including tyrosines 1,221/1,222 and 1,248 in the cytosolic tail of HER2. These phosphotyrosine residues serve as docking sites for SH2-containing and PTB-containing adaptor proteins that link HERs to downstream intracellular signaling cascades, including Ras, extracellular signal-regulated protein (ERK) kinase, phospholipase C gamma, PI3K, Akt and signal transducer and activator of transcription (STAT3)3 pathways.

HER1 undergoes a rapid and pronounced EGF-induced internalization from the plasma membrane as part of a dynamic clathrin-directed trafficking process that plays a key role in regulating its membrane expression, intracellular signaling, and downregulation [10]. HER1 contains canonical motifs that, following its autophosphorylation, directly bind to clathrin and clathrin-associated adaptor proteins. While it has been reported that HER2 co-internalizes with HER1 [11], internalization of HER2 alone from a population of HER2 homodimers has not been observed, even though HER2 contains intracellular motifs that are localized similarly to those present in HER1 [12].

In this report we show that, in comparison with the mAb trastuzumab, exposure of cell-surface HER2 to polyclonal anti-HER2 antibody generated in mice promotes rapid receptor internalization and degradation, accompanied by phosphorylation of the downstream kinasesERK1/2 and Akt. Prolonged exposure to the polyclonal anti-HER2 antibody is characterized by significant HER2 internalization, ubiquitination and degradation, a dramatic reduction in plasma membrane HER2 expression and signaling, and profound anti-tumor activity *in vitro *and *in vivo*. As we reported before, the polyclonal anti-HER2 antibody had a synergistic effect with small-molecule HER2 kinase inhibitors [13]. In addition to antibodies induced by potent adenoviral vectors, we studied HER2-specific antibodies induced by a HER2 protein vaccine being tested in clinical trials in breast cancer patients. The HER2-specific antibodies were detected in the serum of vaccinated patients. These human anti-HER2-specific antibodies were capable of binding to surface expressed HER2 and inhibiting HER2 phosphorylation but did not mediate receptor internalization.

## Materials and methods

### Reagents and antibodies

Vaccine-induced anti-HER2 antibodies (HER2-VIA), LacZ-VIA and GFP-VIA were generated as previously described [13]. Briefly, pooled serum from a large quantity of mice was purified using saturated ammonium sulfate buffer and the concentration of total serum proteins in stock for all of the studies was measured and adjusted to 20 mg/ml in saline.

Trastuzumab was obtained from the Duke University Medical Center Pharmacy (Durham, NC, USA). Neuregulin was purchased from R&D systems (Minneapolis, MN, USA). Mouse and rabbit IgG beads were from eBioscience (San Diego, CA, USA). HER2 antibody 3B5 and ubiquitin antibodies were from Santa Cruz Biotechnology (Santa Cruz, CA, USA). HER2 (29D8), HER3, Akt, pAkt, and surviving antibodies were from Cell Signaling (Beverly, MA, USA). Phospho-specific HER2 antibodies (Y877, Y1221/1222 and Y1248) were also purchased from Cell Signaling. These antibodies were used at 1:500 dilutions in western blotting.

Lamin B1 rabbit antibody was from Abcam (Cambridge, MA, USA). EZ-Link™ Sulfo-NHS-SS Biotin and Streptavidin beads were from Pierce (Rockford, IL, USA). β-actin, *N*-ethylmaleimide, and MG132 were purchased from Sigma (St Louis, MO, USA). Lapatinib was obtained from the Duke University Medical Center Pharmacy.

### Treatment of established HER2-positive human tumor xenografts by passive transfer of vaccine-induced antibodies

These studies were performed under a protocol approved by the Duke University Institutional Animal Care and Use Committee (IACUC) (Durham, NC, USA). Eight-week-old to 10-week-old NOD.CB17-*Prkdc^scid^*/J mice (Jackson Laboratories, Bar Harbor, ME, USA) were implanted with 17β-estradiol pellets (0.72 mg 60-day continuous release pellets; Innovative Research of American, Sarasota, FL, USA) in the flank 1 week prior to the implantation of 5 million BT474M1 tumor cells (kindly provided by Mien-Chie Hung, The University of Texas MD Anderson Cancer Center, Houston, TX, USA). Tumors were allowed to develop for 14 days and then mice were randomized to receive intravenous injection of either GFP-VIA or HER2-VIA (five mice per group). Then 100 to 150 μl vaccine-induced antibodies were injected intravenously at 2-day to 3-day intervals for a total of 10 administrations. Tumor growth was measured in two dimensions using calipers, and the tumor volume was determined using the formula:

$$\text{Volume}=\text{widt}{\text{h}}^{2}\times \text{length}$$

The study was terminated on day 39.

### Flow cytometry analysis

We adapted a methodology reported by Piechocki and colleagues to measure HER2-VIA in vaccinated mouse serum by flow cytometry [14]. Briefly, 3 × 10^5 ^HCC1569 cells were incubated with a mouse anti-human-HER2 mAb or isotype control (Becton Dickinson, San Jose, CA, USA) or with diluted (1:200) mouse serum antibodies (HER2-VIA or LacZ-VIA) for 1 hour at 4°C and then washed with 1% BSA-PBS. The cells exposed to mouse serum containingvaccine-induced antibodies were further stained with phycoerythrin-conjugated anti-mouse IgG (catalogue number R0480; Dako (Carpinteria, CA, USA)) for 30 minutes at 4°C, and washed again. Samples were then analyzed on a Becton Dickinson LSRII flow cytometer with results represented as histograms.

### Cell culture

HEK293 cells were grown at 37°C, 5% CO_2 _in MEM supplemented with 10% fetal bovine serum (Atlanta Biologicals, Lawrenceville, GA, USA), 200 U/ml penicillin, and 50 ng/ml streptomycin (Invitrogen, Carlsbad, CA, USA). SK-BR-3 cells (American Type Culture Collection: HTB-30™) were grown at 37°C, 5% CO_2 _in McCoy's 5A medium supplemented with 10% FCS, 200 U/ml penicillin, and 50 ng/ml streptomycin. HCC1569 cells (American Type Culture Collection: CRL-2330™) were grown at 37°C, 5% CO_2 _in RPM-1640 medium also supplemented with 10% FCS, 1 mM sodium pyruvate, 10 mM Hepes, 0.25% glucose, 200 U/ml penicillin, and 50 ng/ml streptomycin. All cell lines were purchased from American Type Culture Collection (Manassas, VA, USA).

### Construction of fluorescent HER2 construct

HER2-YFP was constructed using a LTR-2/erbB-2(HER2) construct (provided by Dr LE Samelson, NCI, Bethesda, MD, USA) as a PCR template and a pcDNA3.1-mYFP construct as a vector (gift from Roger Y Tsien, University of California at San Diego, USA). HER2 was PCR amplified using the primers 5'-CCCAAGCTTAGCACCATGGAGCTGGCGGCC-3' and 5'-CCGCTCGAGCACTGGCACGTCCAGACCCAG-3', and was inserted into the vector by *Hind*III and *Xho*I restriction sites. HER2 cDNA was verified by sequencing.

### Assay of HER2 endocytosis

Endocytosis of HER2 was assayed using cleavable biotin as described previously [15]. Briefly, SK-BR-3 cells were biotinylated with 1.5 mg/ml sulfo-NHS-SS-biotin (Pierce) in PBS with calcium and magnesium at 4°C for 1 hour. After washing with cold PBS three times, cells were incubated at 37°C for 1 hour in McCoy's 5A medium with or without antibodies (50 µg/ml) to allow endocytosis to occur. Cell-surface biotin was cleaved by incubation (twice, 15 minutes each) in the glutathione cleavage buffer (50 mM glutathione, 75 mM NaCl, 10 mM ethylenediamine tetraacetic acid, 1% BSA, and 0.075 N NaOH). Cells were washed with PBS three times and scraped into the modified RIPA buffer (150 mM NaCl, 50 mM Tris-HCl, pH 7.5, 0.25% (w/v) deoxycholate, 1% NP-40, 5 mM sodium orthovanadate, 2 mM sodium fluoride, and a protease inhibitor cocktail). Cell lysates were incubated with streptavidin beads (Pierce) on a rocking platform overnight at 4°C. After washing, the beads were mixed with the sample buffer and biotinylated proteins were analyzed by immunoblotting using antibodies against HER2.

### Western blotting

The protein samples were subjected to SDS-PAGE using 4-12% Novex^® ^Tris-Glycine Gels (Invitrogen), transferred to nitrocellulose membranes (Bio-Rad Laboratories, Hercules, CA, USA) blocked with 5% nonfat milk powder in TBS-0.2% Tween-20 for 20 minutes, followed by incubation with primary antibodies and then horseradish peroxidase-conjugated secondary antibodies (Amersham Biosciences, Piscataway, NJ, USA). Imaging and quantification of bands were performed using Supersignal (Pierce Biotechnology, Rockford, IL, USA) and AlphaImager™ (Alpha Innotech, Santa Clara, CA, USA).

### Microscopy

HEK293 cells plated on 35-mm, poly-D-lysine-coated, glass-bottom microwell dishes (MatTek Cultureware, Ashland, MA, USA) were allowed to grow for 24 hours and transfected (Fugene6; Roche, Indianapolis, IN, USA) with HER2-YFP construct. Twenty-four hours after transfections, the cells were incubated in culture medium for live cell imaging with 100 µg/ml HER2-VIA, LacZ-VIA, trastuzumab, 20 ng/ml neuregulin or 10 ng/ml EGF. SK-BR-3 and HCC1569 cells plated on the dishes were allowed to grow for 24 hours and were treated as above for the indicated time at 37°C before fixation in 4% paraformaldehyde. Fixed cells were permeabilized and blocked in buffer (5% BSA with 0.2% saponin in PBS) for 20 minutes at room temperature and washed in PBS. Where indicated, cells were incubated with primary antibody (rabbit monoclonal sc-13584; Santa Cruz) in blocking buffer for 1 hour at room temperature, washed, incubated with secondary antibody (Alexa-594-conjugated goat anti-rabbit; Invitrogen) in blocking buffer for 60 minutes at room temperature, washed, and mounted with mounting medium (Vector Laboratories, Inc., Burlingame, CA, USA).

Live cells were studied at 37°C using a heated microscope stage. All slides were examined using a LSM 510-Meta confocal microscope (Carl Zeiss, Thornwood, NY, USA) equipped with 40 × and 100 × apochromat objectives. YFP was excited using a 488-nm argon laser line. Alexa fluorophores were excited at 543 nm using a NeHe laser. Images were processed using the LSM software Image Browser (Carl Zeiss).

### Assay of HER2 degradation and ubiquitination

A total of 0.15 million SK-BR-3 cells or HCC1569 cells plated in six-well plates were allowed to grow for 24 hours in medium with 10% fetal bovine serum and incubated with 100 µg/ml HER2-VIA, LacZ-VIA and trastuzumab in serum-free medium for the indicated time. Cells were washed twice with PBS and lysed with 2 × SDS sample buffer, and the cell lysates were subjected to western blot analysis. For ubiquitination assays, the cells were cultured as described above and then treated with 10 µM MG132 in serum-free medium for 1 hour before incubation with 100 µg/ml HER2-VIA, LacZ-VIA and trastuzumab for the indicated time. After washing twice with PBS, the cells were collected into glycerol lysis buffer (50 mM Hepes, 250 mM NaCl, 0.5% NP40, 10% glycerol, 2 mM ethylenediamine tetraacetic acid) with 5 mM *N*-ethylmaleimide and incubated with 25 µl mouse and rabbit IgG beads for at least 1 hour. The cell lysate was then spun at 18,000 g for 10 minutes to remove the beads, and the supernatant was incubated overnight with 1 µl anti-HER2 antibody 29D8 (Cell Signaling). This was followed by addition of 25 µl rabbit IgG beads for another 2 hours, washing the mix three times with glycerol lysis buffer, and subsequent western blot analysis.

### Patients and treatment/monitoring

The human clinical trial enrolled patients aged 18 or older with stage IV HER2-overexpressing (HER2 3+ or fluorescence *in situ *hybridization-positive) breast cancer who had documented disease progression or relapse following at least one prior standard therapy containing trastuzumab [16]. These patients were immunized with dHER2, a recombinant protein consisting of the extracellular domain and a portion of the intracellular domain of HER2 combined with the adjuvant AS15, containing MPL, QS21, CpG and liposome. Lapatinib (1,250 mg/day) was administered concurrently. Serum was collected at various times pre and post immunization and antibodies were purified from the serum using ammonium sulfate precipitation.

### Statistical analysis

A mixed-effects model was fit to fold-change measurements, with a regression model used to describe the tumor volume by treatment over time. Treatment and mouse were considered categorical variables. Day was considered a continuous variable. Day-squared and the day×treatment and day-squared×treatment interactions were also considered. Treatment and day were considered as fixed effects, and volume measurements for each mouse were considered as repeated measures within treatment. Tumor volume was measured on days 8, 10, 12, 14, 16, 18, and 20 post inoculation. Covariance was modeled as autoregressive. The likelihood ratio test was highly significant (χ^2 ^= 28.27 with 2 degrees of freedom; *P *<0.01), such that one concludes there is a sharp treatment difference in tumor growth over time.

## Results

### Generation of HER2-VIA antibodies against HER2

**Figure 1:**
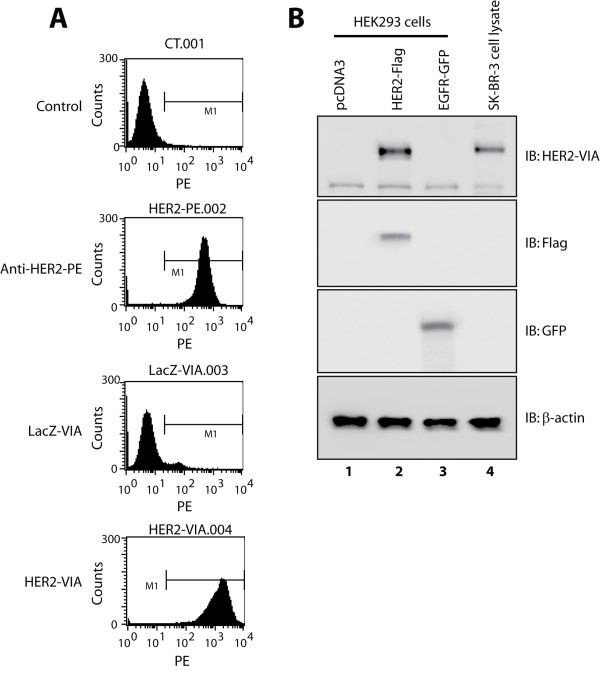
**Characterization of vaccine-induced anti-HER2 antibodies by flow cytometry and western blot analysis**. **(A) **Recognition of cell-surface HER2 by vaccine-induced anti-HER2 antibodies (HER2-VIA). HCC1569 cells were incubated with a mouse anti-human-HER2 mAb (HER2-phycoerythrin (PE)) or isotype control (Becton Dickinson (BD), San Jose, CA, USA), or with diluted (1:200) mouse serum antibodies (HER2-VIA or LacZ-VIA) for 1 hour at 4°C. Samples were then analyzed by BD LSRII flow cytometry, with results represented as histograms. **(B) **Recognition of total HER2 but not epidermal growth factor receptor (EGFR)-GFP by HER2-VIA. HER293 cells expressing vector (lane 1), HER2-FLAG (lane 2) or EGFR-GFP (lane 3) as well as SK-BR-3 cells lysates were western blotted with HER2-VIA (top panel), anti-FLAG antibody (upper middle panel), and anti-GFP antibody (lower middle panel). β-actin was used as a loading control (bottom panel). IB, immunoblot.

We developed adenovirus vectors encoding human HER2/neu with a kinase-inactivating mutation in order to ablate oncogenicity (K753A) [17-21]. The recombinant adenoviruses expressing HER2 were then injected into C57BL/6 mice for anti-HER2 antibody generation. The resulting HER2-VIA were assessed for activity against the extracellular portion of the receptor by flow cytometry, and significant affinity for HER2 at the cell surface was observed (Figure 1A). Based on the flow cytometric assessment of the relative affinity of HER2-VIA compared with trastuzumab in SK-BR-3 cells, we calculated that HER2-VIA-containing crude serum at ~30 μg/ml concentration had the same binding capacity for HER2 as trastuzumab at 1 μg/ml concentration (see Additional file 1). Since the concentration of specific antibodies in crude serum is usually less than 1% of total serum proteins, the affinity of HER2-VIA for HER2 is similar to, if not higher than, that of trastuzumab. Furthermore, overexpressed HER2 or endogenous HER2 protein in SK-BR-3 cells, but not EGFR protein in HEK293 cells, can be detected by HER2-VIA using western blotting, demonstrating the specificity of HER2-VIA for HER2 recognition (Figure 1B).

### Anti-tumor effects of HER2-VIA

**Figure 2:**
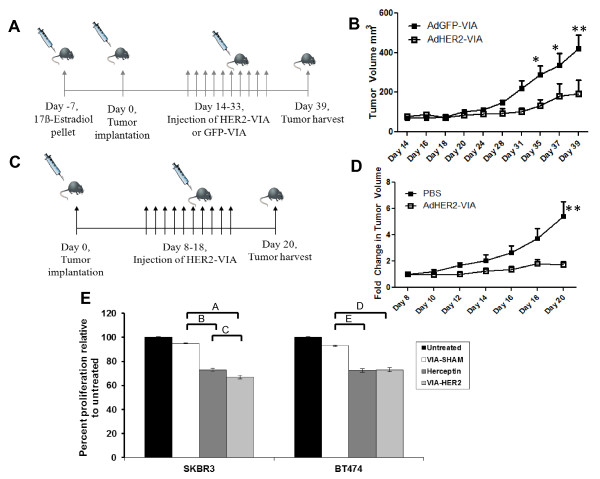
**Vaccine-induced anti-HER2 antibody treatment of established human breast tumor xenografts in mice suppresses tumor growth**. **(A) **Schema for the passive transfer of vaccine-induced antibodies to treat established BT474M1 human breast tumor xenografts in NOD.CB17-*Prkdc^scid^*/J mice. **(B) **Mean tumor volume ± standard error (mm^3^) for AdGFP-VIA-treated (solid squares) and AdHER2-VIA-treated (open squares) mice is presented as a time course from day 14 (initiation of HER2-VIA or GFP-VIA injections) through day 39 (tumor harvest). *P <0.05, **P <0.001. **(C) **Schema for the passive transfer of vaccine-induced antibodies to treat established HCC1569 human breast tumor xenografts in NOD.CB17-*Prkdc^scid^*/J mice. **(D) **Mean fold-change in tumor growth of implanted HCC1569 cells for the mice treated with HER2-VIA or PBS for days 8 through 20. **P <0.001. **(E) **Effect of vaccine-induced anti-HER2 antibodies (HER2-VIA) and trastuzumab on human breast cancer cell proliferation. HER2-positive cells (BT474 or SKBR3) were treated with 3 µl pooled mice crude serum in 100 µl culture medium for 3 days and cell proliferation was assessed by 3-(4,5-dimethylthiazol-2-yl)-2,5-diphenyl tetrazoliumbromide assay. Trastuzumab (Herceptin) (10 µg/ml) was used as a positive control and sera from mice receiving Ad-LacZ vaccine (VIA-SHAM) or saline (untreated) were used as negative controls. Proliferation is plotted relative to the growth of cells in the untreated condition. Error bars represent standard deviation. Data are representative of four experiments. *P *<0.001.

We found that passive immunotherapy with HER2-VIA retards the growth of established HER2-positive human tumor xenografts of BT474M1 cells *in vivo*. Starting at day 21 post inoculation, the average tumor volume in the HER2-VIA-treated group was significantly reduced when compared with mice treated with control GFP-VIA (Figure 2A,B). We also wanted to test the effect of HER2-VIA on tumors expressing HER2 but resistant to trastuzumab and lapatinib. HCC1569 cells are highly resistant to trastuzumab and lapatinib treatment *in vitro*. HER2-VIA was adoptively transferred into HCC1569 tumor-bearing recipient mice and significantly (*P *<0.001) suppressed tumor growth relative to control treated mice (Figure 2C,D, tumor volume fold-change). Accordingly, HER2-VIA - like trastuzumab - inhibits cell proliferation in breast cancer SK-BR-3 and BT474 cells *in vitro*, as shown in Figure 2E. However, HER2-VIA treatment had little effect on HCC1569 cell proliferation *in vitro *(data not shown), suggesting that HER2-VIA functions more effectively against HCC1569 cells *in vivo*. Future studies are needed to determine whether higher concentrations of HER2-VIA are needed to inhibit HCC1569 cells *in vitro*. Based on these anti-tumor effects, we hypothesized that the polyclonal HER2 antibodies may have a direct effect on receptor internalization and degradation. We therefore proceeded to study the molecular events following antibody-receptor interaction.

### Activation of HER2 by HER2-VIA

**Figure 3:**
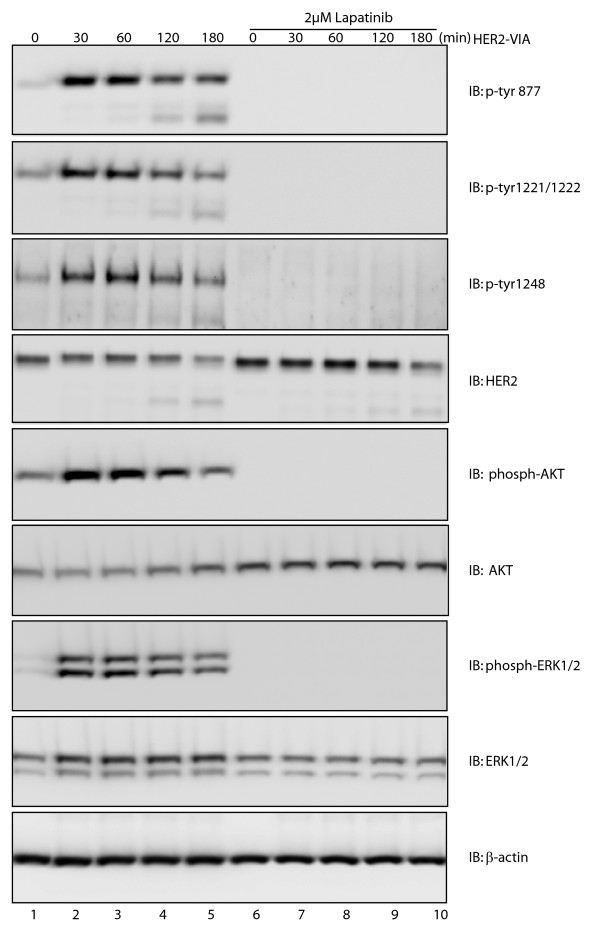
**Activation of multiple signaling molecules by vaccine-induced anti-HER2 antibodies and inhibition of activation by lapatinib**. SK-BR-3 cells were left untreated (lanes 1 to 5) or were pretreated with 2 µM lapatinib for 3 hours (lanes 6 to 10) followed by 100 µg/ml vaccine-induced anti-HER2 antibodies (HER2-VIA) stimulation at 37ºC for the indicated time. After treatment, cells were washed and then lysed in 2 × SDS sample buffer followed by sonication. Equal amounts of protein from each sample were used to visualize the indicated molecules by immunoblotting. ERK, extracellular signal-regulated protein kinase.

Despite a lack of identified HER2-soluble ligands, HER2 phosphorylation on multiple tyrosine sites (including residues 877, 1,221, 1,222 and 1,248) has been established from HER2 activation by homodimerization or heterodimerization [18,22-24]. In SK-BR-3 breast cancer cells transiently stimulated with HER2-VIA, phosphorylation of HER2 on sites 877, 1,221, 1,222 and 1,248, as well as phosphorylation of the downstream HER2 signaling molecules Akt and ERK, was detected (Figure 3, lanes 1 to 5). The HER2 and EGFR tyrosine kinase inhibitor lapatinib abolished tyrosine phosphorylation at these sites, as well as blocking activation of Akt and ERK (Figure 3, lanes 6 to 10). These data indicate that HER2-VIA initially behaves as a HER2 agonist, similar to trastuzumab [25-27].

### Internalization of HER2 upon HER2-VIA stimulation

**Figure 4:**
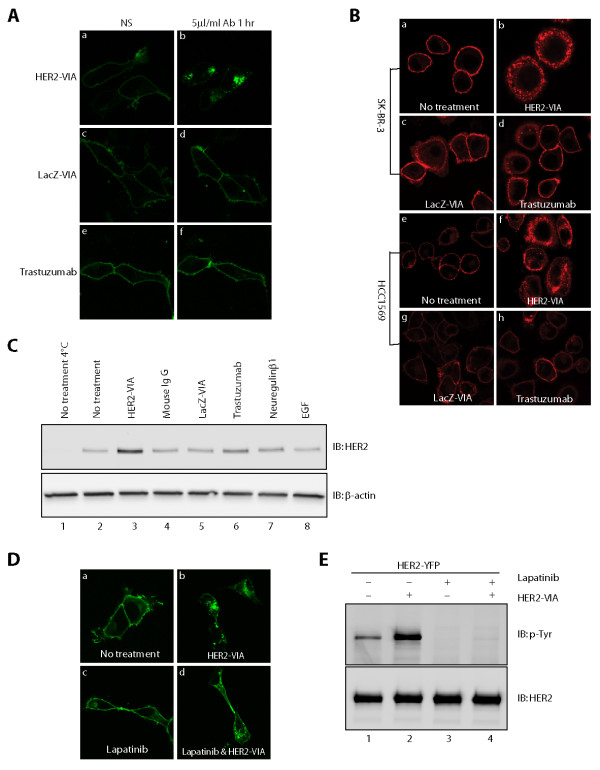
**Effects of vaccine-induced anti-HER2 antibodies on HER2 internalization**. **(A) **Internalization of HER2-YFP by vaccine-induced anti-HER2 antibodies (HER2-VIA). HEK 293 cells transiently expressing HER2-YFP were stimulated with **(a, b) **HER2-VIA, **(c, d) **LacZ-VIA, or **(e, f) **trastuzumab, respectively for 1 hour. Confocal images from the same cells were taken before and after antibody incubation. Formation of vesicles in the cells indicated receptor internalization. The experiment was repeated three times. NS, non-stimulated. **(B)**. Imaging endogenous HER2 internalization by HER2-VIA. SK-BR-3 and HCC1569 cells were allowed to grow for 24 hours and were treated as described in Materials and methods. Confocal images of cells that **(a, e) **were left untreated, or were treated with **(b, f) **HER2-VIA, **(c, g) **LacZ-VIA or **(d, h) **trastuzumab. **(C) **Endogenous HER2 internalization by HER2-VIA as assessed by cell surface Biotin-labeling. Upper panel: immunoblot (IB) for protected (internalized) biotin-labeled HER2 in cells treated with indicated agents/conditions. Lower panel: β-actin serves as loading control to ensure equal amounts of cell lysate. EGF, epidermal growth factor. **(D) **Effect of lapatinib on HER2-YFP internalization induced by HER2-VIA. Confocal images of HEK293 cells expressing HER2-YFP that **(a) **were left untreated, or were treated with **(b) **HER2-VIA, **(c) **lapatinib or **(d) **lapatinib then HER2-VIA for 1 hour. **(E) **The same transfected cells were immunoblotted (IB) for HER2 tyrosine phosphorylation (upper panel) and total HER2 expression (lower panel).

To characterize the effect of antibodies on HER2 receptor internalization, HEK293 cells transfected with HER2-YFP were incubated with 100 µg/ml HER2-VIA antibody for 1 hour prior to observation. HER2-VIA-induced internalization of HER2-YFP resulted in the formation of fluorescent cytosolic aggregates (Figure 4A, a vs. b). As shown in Additional file 1, 100 µg/ml HER2-VIA antibody exhibits a HER2 fluorescence staining intensity comparable with 5 µg/ml trastuzumab. However, a 20-fold excess of trastuzumab (100 µg/ml) behaved similarly to LacZ-negative control antibody and was unable to internalize receptor, because YFP fluorescence remained at the plasma membrane (Figure 4A, c to 4f).

We next sought to confirm the findings of Figure 4A for endogenous HER2 receptor internalization using breast cancer cell lines SK-BR-3 and HCC1569, which each express abundant HER2 protein but are sensitive or resistant to trastuzumab treatment, respectively [28]. As is shown (Figure 4B, a to 4c), HER2-VIA induced HER2 internalization in SK-BR-3 cells while LacZ control antibody had little to no effect. Trastuzumab produced only a small complement of internalized HER2 receptors in SK-BR-3 cells (Figure 4B, d). Similarly, treatment of HCC1569 cells with HER2-VIA caused a significant amount of endogenous HER2 internalization while LacZ-VIA and trastuzumab had no effect (Figure 4B, e to 4h). Counterstaining cells with the nuclear membrane marker lamin B indicates that the internalized endogenous HER2 localizes exclusively to the cytosol (see Additional file 2), suggesting that HER2 internalization does not lead to HER2 nuclear translocation as reported [29].

We further studied the effect of antibodies on endogenous HER2 receptor endocytosis using a biotin method to label HER2 at the cell surface, as shown in Figure 4C. Western blotting of biotin-labeled internalized receptors that originated at the cell surface demonstrates that HER2-VIA induces robust HER2 internalization (Figure 4C, lane 3 vs. rest of the lanes). Note also that raising the temperature of the cells from 4 to 37ºC induces a small amount of HER2 internalization (Figure 4C, lanes 1 and 2) [15]. In addition, HER2 internalization induced by HER2-VIA is tyrosine kinase independent, because it occurs in the presence of the kinase inhibitor lapatinib (Figure 4D,E).

### Internalization of HER2 through clathrin-coated pits/vesicles

**Figure 5:**
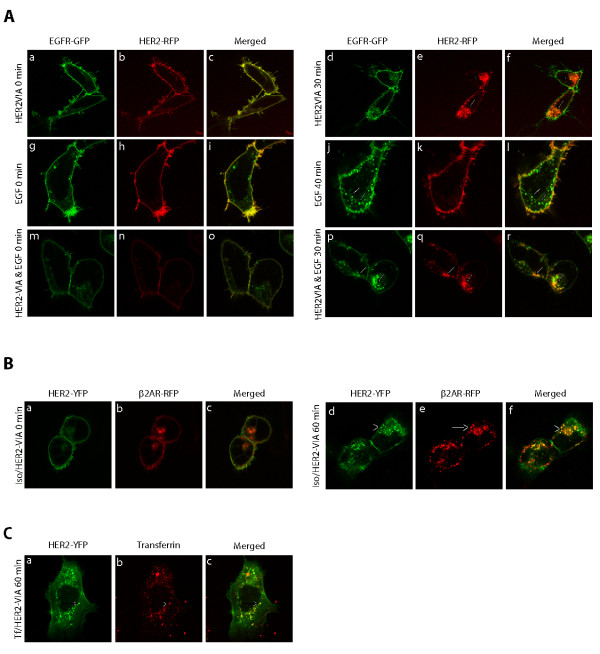
**HER2 internalizes through a clathrin-coated mechanism**. **(A) **Effect of HER2 and epidermal growth factor receptor (EGFR) trafficking by vaccine-induced anti-HER2 antibodies (HER2-VIA) and epidermal growth factor (EGF). HEK 293 cells transiently expressing HER2-RFP and EGFR-GFP were **(a to c, g to i, m to o) **left untreated, or were stimulated with **(d to f) **HER2-VIA, **(j to l) **EGF and **(p to r) **HER2-VIA/EGF at 37°C for 30 minutes. Confocal images from the same cells were taken before and after HER2-VIA and/or EGF ligand stimulation. Arrowheads indicate internalized receptors. Representative images from three experiments are presented. **(B) **Co-localization of HER2-VIA-stimulated HER2-YFP with the β_2_-adrenergic receptor (β_2_AR). Confocal images of unstimulated HEK293 cells expressing both **(a) **HER2-YFP and **(b) **β_2_AR-RFP. **(c) **Merged image of (a) and (b). **(d to f) **Confocal images of cells simultaneously stimulated with 0.1 μM isoproterenol (Iso) and 100 μg/ml HER2-VIA at 37°C for 1 hour. **(f) **Merged image of (d) and (e). Arrowheads indicate co-localized vesicles. **(C) **Co-localization of HER2-VIA-stimulated HER2-YFP with transferrin. **(a to c) **Confocal images of cells expressing the HER2-YFP and treated simultaneously with Alexa-546 transferrin (Tf) (100 μg/ml) and HER2-VIA (100 µg/ml) for 1 hour at 37°C. (c) Merged picture. Arrowheads indicate co-localized vesicles.

Upon EGF stimulation, HER1 (EGFR) undergoes internalization through clathrin-coated pits/vesicles where it co-internalizes with HER2 [30-32]. To elucidate the HER2 internalization process, we examined the capacity of HER2-VIA to modulate trafficking of heterodimers of EGFR and HER2 (Figure 5A). GFP protein was fused to the C-terminus of EGFR (EGFR-GFP) and RFP protein was fused to the C terminus of HER2 (HER2-RFP). In addition to membrane localization, a small percentage of the EGR receptors were localized to the cytoplasm basally when co-expressed with HER2 in HEK293 cells (Figure 5A, a, g and 5m). This is probably due to the constitutive endocytosis and recycling of EGFRs that is known to be ligand independent [33,34]. HER2-VIA treatment of HEK293 cells transiently co-expressing HER1-GFP (EGFR-GFP) and HER2-RFP caused robust HER2-RFP internalization (Figure 5A, a to 5f). However, the overall distribution and the membrane localized EGFR were not altered by the HER2-VIA treatment, suggesting that the HER2-VIA-induced endocytosis is specific for HER2 (Figure 5A, d). In contrast, EGF ligand treatment caused robust EGFR-GFP internalization but had little or no effect on HER2 distribution (Figure 5A, g to 5l). When cells were treated simultaneously with HER2-VIA and EGF, internalization of both HER2 and EGFR was observed and the two markers extensively overlapped within the cells (Figure 5A, m to 5r). These data are also suggestive of HER2 utilizing the same clathrin mechanism for receptor internalization as EGFR [10].

β_2_-adrenergic receptors are prototypical for clathrin-dependent internalization of G-protein-coupled receptors [35,36], and transferrin is a well-documented standard for clathrin-mediated internalization in general [37]. The demonstration that internalized HER2-YFP co-localizes with either β_2_-adrenergic receptor-RFP or transferrin in intracellular vesicles would further confirm that HER2 also internalizes in manner similar to these other proteins. As shown in Figure 5B, in cells expressing both β_2_-adrenergic receptor-RFP and HER2-YFP receptors and prior to activation, the two receptors are not intracellularly co-localized (Figure 5B, a to 5c). Exposure to isoproterenol and HER2-VIA for 1 hour resulted in multiple overlapping intracellular distributions of these receptors (Figure 5B, d to 5f). Similarly, internalized transferrin at 1 hour has significant co-localization with internalized HER2-YFP (Figure 5C). To further demonstrate that HER2-VIA-induced HER2 internalization occurs through clathrin-coated pits, SK-BR-3 cells were treated with sucrose, a known inhibitor of clathrin-dependent endocytosis [38]. As shown in Additional file 3, HER2-VIA-induced internalization of HER2 was effectively blocked by sucrose, and this inhibition was reversible because removing sucrose from the cells allowed the internalization of HER2 to proceed. Taken together, these data suggest that HER2-VIA-induced HER2 internalization occurs through a clathrin-coated pit mechanism.

### Ubiquitination of HER2 upon HER2-VIA stimulation

**Figure 6:**
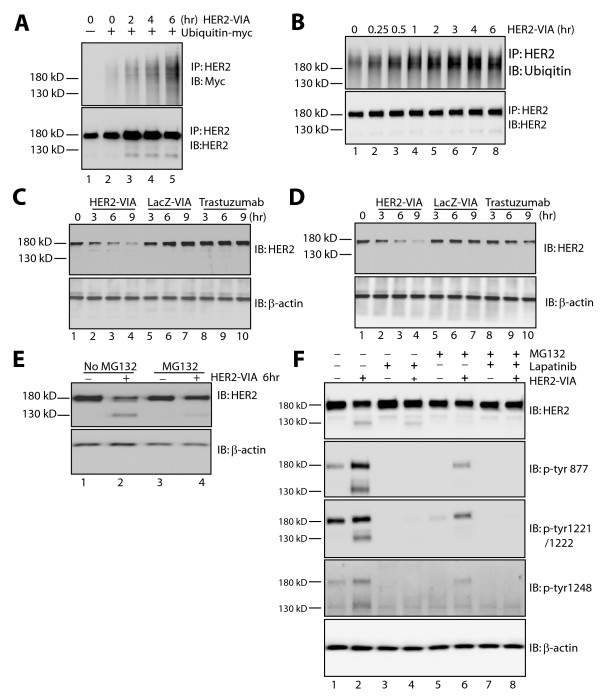
**HER2 ubiquitination, degradation, and fragmentation induced by vaccine-induced anti-HER2 antibodies**. **(A) **Vaccine-induced anti-HER2 antibodies (HER2-VIA) stimulation led to HER2 ubiquitination. SK-BR-3 cells expressing ubiquitin-Myc were pretreated with MG132 for 2 hours and then treated with HER2-VIA for the indicated time. The ubiquitinated HER2 was detected by anti-Myc antibody. **(B) **HER2-VIA stimulation leads to HER2 ubiquitination. SK-BR-3 cells were pretreated with MG132 for 2 hours before HER2-VIA stimulation. Cell lysates were immunoprecipitated with anti-HER2 29D8 antibody. The endogenous ubiquitinated HER2 was detected by the anti-ubiquitin antibody and the total HER2 was visualized by anti-HER2 3B5 antibody. **(C), (D) **HER2-VIA stimulation causes HER2 degradation: (C) SK-BR-3 and (D) HCC1569 cells were stimulated with HER2-VIA, LacZ-VIA, or trastuzumab for the indicated time, and an equal amount of cell lysates was subjected to western blot analysis. Expression levels of HER2 and β-actin were detected by corresponding antibodies. **(E) **HER2-VIA stimulation produces HER2 fragmentation. SK-BR-3 cells were treated with HER2-VIA for 6 hours in the absence or presence of prior MG132 treatment for 2 hours. Full-length and truncated HER2 are detected by anti-HER2 3B5 antibody. β-actin serves as loading control. **(F) **HER2-VIA stimulation produces tyrosine phosphorylation of the 130 kDa HER2 C-terminal fragment. SK-BR-3 cells were incubated with HER2-VIA for 6 hours after pretreatment with lapatinib, MG132, or lapatinib plus MG132 for 2 hours. Full-length and truncated HER2 are detected by anti-HER2 3B5 antibody, which recognizes the C-terminus (top panel). Phosphorylated full-length and truncated HER2 were recognized by tyrosine site-specific phospho-antibodies for phosphorylated tyrosine 877, 1,221/1,222 and 1,248 (middle three panels). IB, immunoblot.

Receptor internalization is often associated with receptor ubiquitination and targeting to proteasomes for degradation. In SK-BR-3 cells transiently expressing exogenous Myc-tagged ubiquitin, HER2-VIA treatment resulted in increased HER2-ubiquitin-Myc in the presence of 10 µM of the protease inhibitor MG132, as assessed by western blotting (Figure 6A). HER2-VIA also led to ubiquitination of endogenous HER2 receptor in SK-BR-3 cells in the presence of MG132 (Figure 6B). Interestingly, we found that the ubiquitination of HER2 induced by HER2-VIA treatment was effectively blocked by 1 µM lapatinib (see Additional file 4). It has also been shown previously that lapatinib is capable of stabilizing HER2 proteins by inhibiting basal-induced and trastuzumab-induced ubiquitination of HER2 [26]. Our results are consistent with the notion that HER-VIA functions by activating HER2 receptors and subsequently triggers internalization and ubiquitination.

### Degradation of HER2 upon HER2-VIA stimulation

To explore the stability of HER2 after internalization, SK-BR-3 cells were incubated with HER2-VIA and harvested. Using western blot analysis we demonstrated that HER2-VIA treatment for 3 hours induced a significant reduction of total HER2 (Figure 6C). A 9-hour treatment reduced HER2 protein expression by about 70%. In contrast, the amount of HER2 protein did not decrease after treatment with control antibody LacZ-VIA. Trastuzumab also did not reduce HER2 protein expression (Figure 6C). Similar results were obtained in HCC1569 cells (Figure 6D), suggesting that HER2-VIA is an effective antibody for promoting HER2 degradation.

### Truncation of HER2 upon HER2-VIA stimulation

HER2 is a 1,255-amino-acid protein that migrates at 185 kDa. Similar to other EGF family receptors, the extracellular domain of HER2 can be cleaved, reportedly at amino acid site R647, A644 or N530 [39]. Following HER2-VIA stimulation we observed the formation of a 130 kDa fragment of HER2 that was associated with a reduction in full-length HER2 expression (Figure 6E). Blockade of proteasome digestion prevented the appearance of this cleaved form of HER2 (Figure 6E). Moreover, this fragment was phosphorylated at tyrosine residues (referenced to full-length HER2) 877, 1,221, 1,222 and 1,248 as detected by phospho-specific antibodies (Figure 6F, lanes 1 and 2). Since tyrosine 1,248 is present in this 130 kDa fragment, the cleavage must take place at the amino-terminal end of HER2. Lapatinib treatment had no effect on the appearance of the 130 kDa fragment (Figure 6F, lanes 3 and 4) while the protease inhibitor MG132 blocked its formation (Figure 6F, lanes 5 and 6). However, HER2-VIA-induced tyrosine phosphorylation of the fragment was abolished with lapatinib treatment (Figure 6F, lanes 3 and 4). Taken together, these data suggest that this 130 kDa fragment is cleaved from the HER2 N-terminus and may represent a novel cleavage site, distinct from those observed previously [40] (Figure 6E).

### Reduced signaling by HER2 following prolonged-HER2-VIA treatment

**Figure 7:**
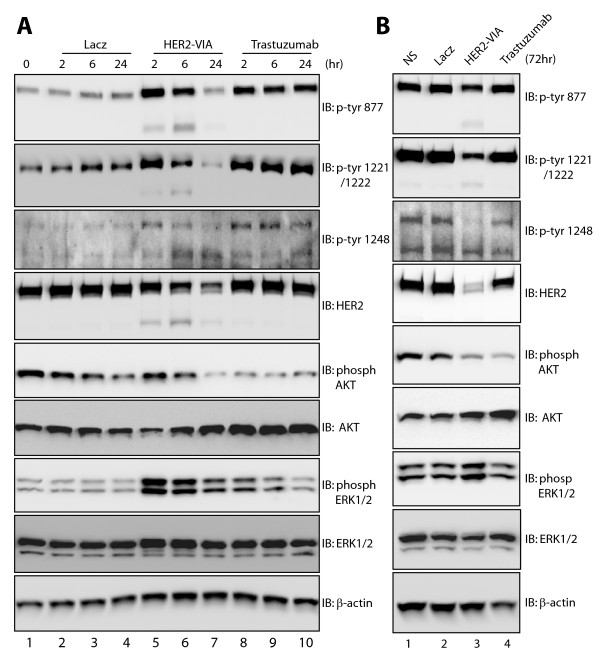
**Reduced signaling by HER2 following prolonged vaccine-induced anti-HER2 antibody treatment**. SK-BR-3 cells were stimulated for **(A) **24 hours and **(B) **72 hours with LacZ-VIA (A, lanes 2 to 4; B, lane 2), HER2-VIA (A, lanes 5 to 7; B, lane 3), and trastuzumab (A, lane 8 and 9; B, lane 4). Protein samples were immunoblotted with indicated antibodies. β-actin in the lower panel serves as a loading control. *n *= 3. ERK, extracellular signal-regulated protein kinase; IB, immunoblot; NS, non-stimulated.

In SK-BR-3 breast cancer cells transiently exposed to HER2-VIA, phosphorylation of HER2 on sites 877, 1,221, 1,222, and 1,248 as well as phosphorylation of the downstream HER2 signaling molecules AKT and ERK was detected (Figure 3, lanes 1 to 5). However, prolonged HER2-VIA binding (up to 72 hours stimulation) resulted in decreased AKT phosphorylation, probably due to the decreased HER2 expression after prolonged treatment (Figure 7A,B).

### Effects of human HER2-specific antibodies

As an extension of the above experiments, we were interested in studying the signaling effects of human antibodies generated against HER2 in the setting of tyrosine kinase inhibition, Prior to utilizing our recombinant viral vectors expressing HER2 with lapatinib in humans, important safety issues regarding combination HER2-specific vaccination and kinase inhibition needed assessment. We therefore proceeded to study the concept of vaccination with concomitant kinase inhibition using a HER2 protein vaccine (dHER2) that was in clinical trial development in combination with lapatinib for this first study in humans. The vaccine we proposed to test, dHER2, consists of the extracellular domain and part of the intracellular domain of HER2 combined with the adjuvant AS15 containing MPL, QS21, CpG and liposome.

**Figure 8:**
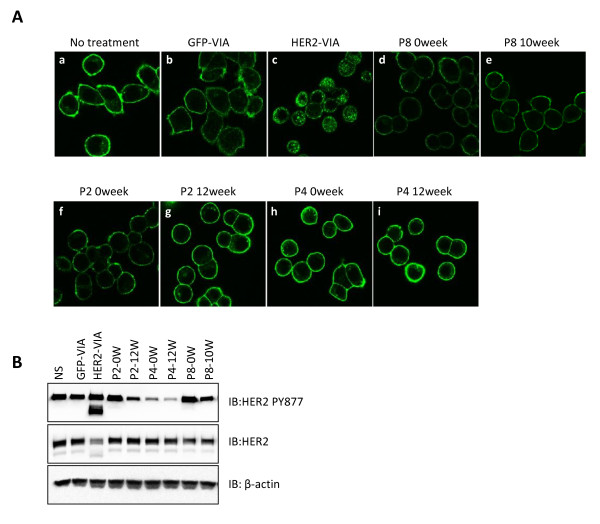
**Reduced signaling of HER2 following patient's serum treatment**. **(A) **Imaging of endogenous HER2 internalization by the human HER2-specific antibodies and vaccine-induced anti-HER2 antibodies (HER2-VIA). All human serum samples were purified to deplete lapatinib prior to using in the internalization assay. SK-BR-3 cells were allowed to grow for 24 hours and were treated for 1 hour with different murine or human antibodies as indicated above each image. Following treatment, the cells were fixed and stained to visualize the cellular distribution of HER2 using HER2 antibody 29D8 as described in Materials and methods. Confocal images of cells that **(a) **were left untreated, or were treated with **(b) **control GFP-VIA, **(c) **HER2-VIA, **(d, e) **serum (week 0 and week 10) from Patient 8, **(f, g) **serum (week 0 and week 12) from Patient 2, and **(h, i) **serum (week 0 and week 12) from Patient 4. **(B) **Effect of human HER2-specific antibodies on Her2 tyrosine 877 phosphorylation. SK-BR-3 cells were stimulated for 9 hours with indicated sera from either patients or mice. Protein samples were immunoblotted with anti-Her2PY877 antibodies (upper panel), anti-Her2 antibodies (middle panel), or anti-β-actin antibodies as a loading control (lower panel). *n *= 3. IB, immunoblot; NS, non-stimulated.

In this study, women with metastatic, trastuzumab-refractory HER2-overexpressing breast cancer were immunized six times at 2-week intervals with dHER2 concomitantly with oral lapatinib (1,250 mg/day). The clinical results and primary immune analysis of this study are reported elsewhere [16]. The specificity of human serum samples against HER2 receptors has been verified using ELISAs as described in the previous study [16]. To further determine the molecular mechanism of human HER2 antibodies, crude serum antibodies from three patients with the highest titer of antibodies to the HER2 extracellular domain were obtained via ammonium sulfate precipitation to deplete lapatinib and then tested for binding to HER2-expressing cells. Although human HER2 antibodies are capable of binding to HER2 as shown previously [16], no receptor internalization was observed after 1 hour of incubation in contrast to the rapid receptor internalization and degradation noted with the murine HER2-VIA (Figure 8A). Even after 6 hours of treatment, no HER2 internalization was observed in SK-BR-3 cells incubated with human antibodies (data not shown). Nonetheless, phosphorylation of HER2 (tyrosine 877) was markedly decreased by patients' serum upon 6 hours of treatment (Figure 8B); meanwhile, the phosphorylation of HER2 (tyrosine 877) remained relatively unchanged upon short-term stimulation of SK-BR-3 with patient antibodies, unlike murine HER2-VIA, which possesses agonistic effect on HER2 signaling (see Additional file 5).

In summary, our studies suggest that HER2-VIA antibodies possess some properties characteristic of agonists, such as promoting HER2 signaling, but that this is soon followed by receptor internalization, ubiquitination and, finally, degradation and downregulation of signaling.

## Discussion

Constitutive HER2 internalization and membrane recycling normally occur at a very slow rate in many cell systems in the absence of a recognized agonist. A recent report indicates that one mAb or multiple mAbs against HER2, alone or in combination, were able to induce HER2 internalization over intervals as short as 4 hours, but the mechanisms underlying the internalization were not determined [41]. Interestingly, even slow HER2 internalization is associated with reductions in HER2 signaling and decreases in tumor growth rates. We found that polyclonal anti-HER2 antibodies were remarkably more potent than the mAb trastuzumab in causing HER2 internalization and degradation. Our data indicate that HER2-VIA antibodies are HER2 agonists that bind and internalize the receptor, signal through ERK1/2 and Akt, and deplete HER2 from the plasma membrane by the same clathrin-mediated mechanisms utilized by other HER family members exposed to cognate agonists.

Although polyclonal antibodies specific to HER2 are not clinically available, active immunotherapy targeting tumor antigens can lead to tumor antigen-specific immune responses. We have administered HER2 protein-loaded autologous dendritic cells to patients with advanced HER2-overexpressing tumors. Remarkably, we found that all patients were long-term survivors, and six out of seven patients had developed HER2-specific antibodies that inhibited tumor growth *in vitro *[13]. Alternatives to autologous dendritic cell vaccines include tumor antigen-expressing recombinant vectors. We have developed a potent novel recombinant vector expressing kinase-inactivated HER2 that induced high levels of HER2-specific antibodies [17], and vaccination with this vaccine demonstrated synergy when combined with HER2 kinase inhibition with lapatinib [13], consistent with clinical observations that combinations of antibodies and small-molecule tyrosine kinase inhibitor are more effective than monotherapy.

The HER2-VIA binding sites that are both necessary and sufficient for rapid HER2 removal from the plasma membrane remain unidentified, but these sites could become future targets for small molecules or could be important to elucidation of structure-activity relationships. Other agents are known to produce HER2 internalization but with slower kinetics, suggesting that allosteric modulation of receptor conformation may be sufficient to achieve desired therapeutic goals in the absence of an identified HER2 agonist. Exposure of cells to the ansamysin antibiotic geldanamycin results in HER2 degradation with cleavage of a 130 kDa C-terminus fragment, the separation of HER2 from Hsp90, and within 2 hours the accumulation of HER2 in cytoplasmic vesicles [40]. It is unclear whether the vesicular compartmentalization of HER2 is a consequence of enhanced receptor internalization or an inhibition of receptor recycling, but recent evidence points to the latter. It also has not been determined whether geldanamycin leads to HER2 activation or whether HER2 trafficking is clathrin dependent or independent, asboth explanations have been proposed [42-44].

Interestingly, our western blotting data indicate that the majority of HER2 receptors remain intact following HER2-VIA treatment. However, in contrast to geldanamycin treatment, a 130 kDa fragment truncated at the N-terminus of HER2 is observed in cells that are treated with HER2-VIA, while mAb treatment does not produce a similar fragment. Moreover, we show that HER2-VIA-induced internalization of HER2 receptors precedes HER2 degradation. The internalized HER2-containing vesicles (observed in Figure 4A, b) may represent the receptors localized in the endosome compartment, where the receptors are waiting to be further sorted and can still signal. The level of HER2 remains relatively unchanged until the receptors traffic to the lysosome at a later time point where the protein degradation occurs. Our finding is consistent with a recent study showing that geldanamycin-induced HER2 internalization can be observed within 2 hours whereas the degradation of HER2 did not occur until 6 hours after the treatment [45]. The half-life of HER2 in HER2-VIA-treated cells is around 3 hours, which is similar to that observed in geldanamycin-treated cells [40]. Interestingly, the time course of Akt dephosphorylation tightly correlates with the time course of HER2-VIA-induced dephosphorylation and degradation of HER2. In HER2-overexpressing cells such as SK-BR-3, the termination of downstream signaling probably only occurs after the level of HER2 is reduced substantially. Taken together, our findings indicate that although HER2-VIA shares certain similarities with trastuzumab and geldanamycin in its ability to inhibit HER signaling, it affects HER2 behavior in a manner quite distinct from either trastuzumab or geldanamycin.

The responses we observed with polyclonal anti-HER2 antibodies suggest that their mechanism of action may underlie new strategies for cancer immunotherapy. Most active immunotherapy strategies have not resulted in large clinical reductions of tumors. Nonetheless, recent reports have suggested that, despite the absence of classical clinical responses to active immunotherapy, there have been long-term survival benefits [46,47]. We anticipated that the generation of HER2-specific antibodies in patients with breast cancer would have similar effects to the murine HER2-specific antibodies. While we were preparing our recombinant HER2-expressing adenoviral vaccine for human testing, we performed a pilot study of vaccination of breast cancer patients with a combination of HER2 protein vaccine and lapatinib. Although this vaccine induced detectable HER2-specific antibodies that could recognize HER2 expressed on the surface of tumor cells, we did not see receptor internalization and degradation. We were able to document that the antibodies had an inhibitory effect on HER2 signaling *in vitro*, as had been previously reported with peptide-based vaccines. Nonetheless, more potent vaccines targeting HER2 may be capable of generating higher-titer HER2-specific antibodies, and antibodies that not only bind but mediate receptor internalization and degradation, and the resultant loss of HER2 signaling.

Our studies provide new insights into the mechanisms underlying HER2 receptor trafficking and provide proof-of-principle that HER2 can be rapidly removed from the cell surface by agonist-like mimetics that have agonist effects. The absence of HER2 agonists has impeded development of therapies that exploit the relationship between plasma membrane HER2 expression and inappropriate HER2 signaling, but our findings suggest a long-term clinical benefit from oncogenic signaling ablation. Our results with HER2-VIA provide a basis for developing new classes of HER2 signaling inhibitors for patients that are resistant to current modes of therapy. These data also support the clinical evaluation of cancer vaccine strategies targeting HER2, with overall survival rather than tumor shrinkage/progression-free survival as an endpoint.

## Conclusions

We have found that polyclonal anti-HER2 antibodies (HER2-VIA) generated by vaccinating mice with an adenovirus expressing human HER2 can retard the growth of established HER2-positive human tumor xenografts *in vivo*, bind to HER2 at the plasma membrane, induce HER2 internalization, ubiquitination and degradation, and eventually inactivate downstream kinase Akt. We have also demonstrated that low-titer HER2-specific antibodies induced by vaccinating breast cancer patients with a HER2 protein vaccine can bind to receptor and inhibit HER2 signaling through blocking tyrosine 877 phosphorylation of HER2, but did not induce receptor internalization and degradation. These data support the testing of more potent HER2-specific vaccines in human clinical trials.

## Abbreviations

BSA, bovine serum albumin; EGF, epidermal growth factor; EGFR, epidermal growth factor receptor; ERK, extracellular signal-regulated protein kinase; FCS, fetal calf serum; GFP, green fluorescent protein; HER, human epidermal growth factor receptor; HER2-VIA, vaccine-induced anti-HER2 antibodies; mAb, monoclonal antibody; MEM, modified Eagle's medium; PBS, phosphate-buffered saline; PCR, polymerase chain reaction; RFP, red fluorescence protein; YFP, yellow fluorescent protein.

## Competing interests

EH, KB, AH, NS, HKL and MAM received financial support from GSK to support the research described in the manuscript. TC is employed by and/or a stockholder of GSK. The remaining authors declare that they have no competing interests.

## Authors' contributions

X-RR participated in the design and coordination of the study, data acquisition and analysis, and helped draft the manuscript. JuW participated in the data acquisition and analysis. GL participated in the data acquisition and analysis. JiW participated in the coordination of the study and helped draft the manuscript. JL participated in the coordination of the study and helped draft the manuscript. WX participated in the design of the study and helped draft the manuscript. NS participated in the design of the study and helped draft the manuscript. LB participated in the design of the study and helped draft the manuscript. TC participated in the design of the study and helped draft the manuscript. TO helped with manuscript revision. EH participated in the design of the study, participated in the recruitment of patients, managed specimen acquisition and preparation from the human study, and helped draft the manuscript. KB participated in the recruitment of patients and helped draft the manuscript. AH participated in the coordination of the study, immune analysis of human specimens, and helped draft the manuscript. MAM participated in the design the study, managed specimen acquisition and preparation from the human study and helped draft the manuscript. HKL participated in the design and coordination of the study, and help draft the manuscript. WC participated in the overall design and coordination of the study and drafted the manuscript. All authors gave final approval of the manuscript for publication.

## Supplementary Material

Additional file 1 Figure S1 showing flow cytometric assessment of the relative HER2-VIA and trastuzumab binding intensity to HER2-positive SK-BR-3 human breast tumor cells. SK-BR-3 cells were incubated with the indicated dilution of **(A) **HER2-VIA (1:100 to 1:102,400) or **(B) **trastuzumab (20 to 0.02 μg/ml) and then stained with the appropriate phycoerythrin-conjugated anti-IgG secondary antibody. Mean fluorescence intensity, as a measure of antibody binding to HER2, was plotted. LacZ-VIA as a negative control showed an MFI (mean fluorescence intensity) of less than 50 at all dilutions (1:100 to 1:102,400) (data not shown). Click here for file

Additional file 2 Figure S2 showing HER2-VIA drives HER internalization to the cytoplasm but not to the nucleus. Confocal images of SK-BR-3 cells were **(a to c) **left untreated, or **(d to f) **treated with HER2-VIA for 60 minutes. Cells were stained with anti-HER2 antibody (a, d) and lamin B (b, e). (c, f) Merged pictures. Click here for file

Additional file 3 Figure S3 showing sucrose inhibits HER2-VIA-induced internalization of HER2. SK-BR-3 cells were treated with 20 µl HER2-VIA and then incubated with FITC 488-conjugated goat anti-mouse antibody on ice. The cells were then exposed to the following conditions and were then imaged by confocal microscopy: **(a) **incubation on ice for 1 hour; **(b) **incubation at 37°C for 1 hour; **(c) **incubation in 0.45 M sucrose on ice; **(d) **incubation in 0.45 M sucrose at 37°C for 1 hour; **(e) **incubation in 0.45 M sucrose on ice for 30 minutes followed by washing and then incubation of the cells at 37°C for 1 hour. Click here for file

Additional file 4 **Figure S4 showing inhibition of HER2-VIA-induced HER2 ubiquitination by lapatinib**. SK-BR-3 cells were pretreated with the proteasome inhibitor MG132 (10 µM) and lapatinib for 30 minutes before HER2-VIA application for 2 hours. After the indicated treatment, cells were lysed and HER2 was precipitated using anti-HER2 rabbit antibody 29D8. Precipitated proteins were subjected to western blot analysis. Upper panel: ubiquitinated HER2; lower panel: total HER2 visualized by anti-HER2 rabbit antibody 29D8. Click here for file

Additional file 5 **Figure S5 showing the effect of human HER2-specific antibodies on HER2 tyrosine 877 phosphorylation**. SK-BR-3 cells were stimulated for 1 hour with the indicated sera from either patients or mice. Protein samples were immunoblotted with anti-HER2 PY877 antibodies (upper panel) or anti-HER2 antibodies (lower panel). Click here for file
