# Supplementary figures and images for: Polyclonal HER2-specific antibodies induced by vaccination mediate receptor internalization and degradation in tumor cells
Source: Breast Cancer Res. 2012 Jun 7;14(3):R89. doi: 10.1186/bcr3204 (PMC3446352; doi:10.1186/bcr3204)

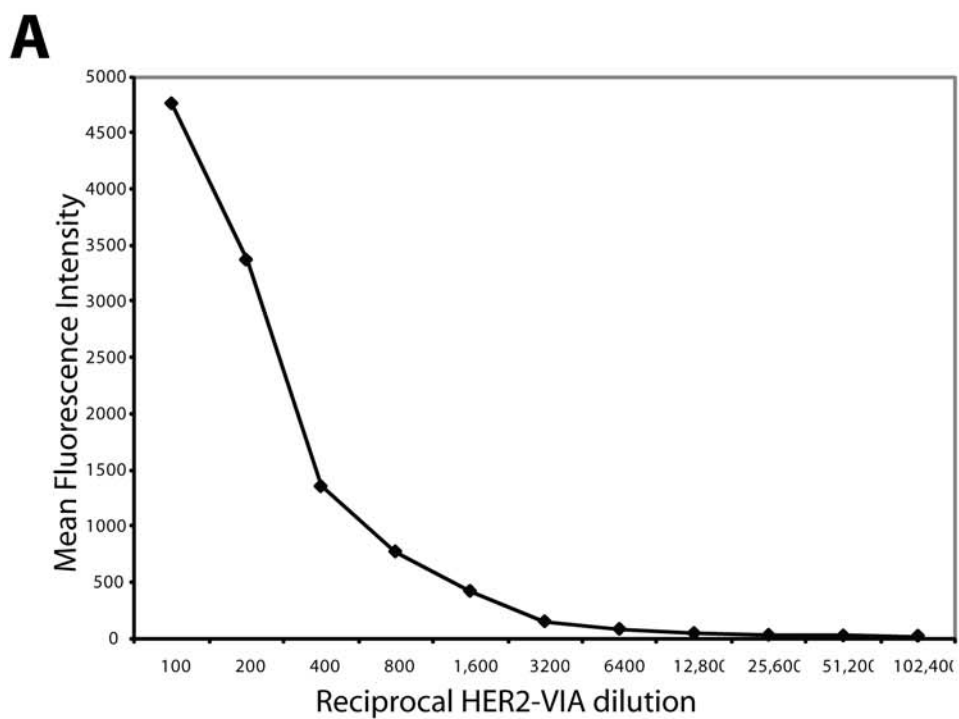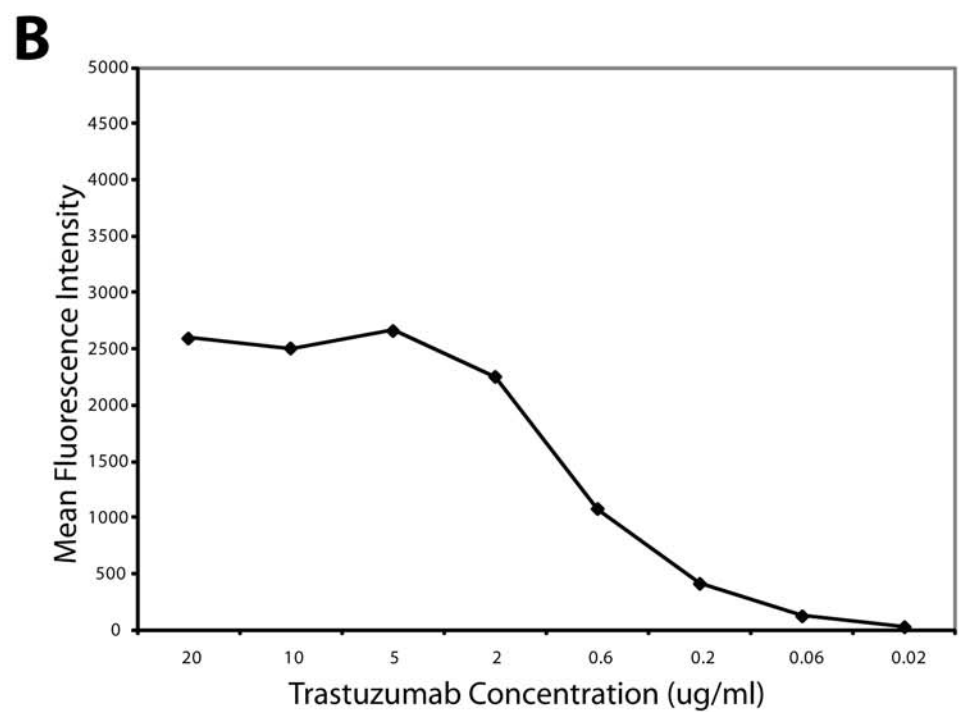

**Supplemental figure 1**

Supplement: Additional file 1 — Figure S1 showing flow cytometric assessment of the relative HER2-VIA and trastuzumab binding intensity to HER2-positive SK-BR-3 human breast tumor cells. SK-BR-3 cells were incubated with the indicated dilution of (A) HER2-VIA (1:100 to 1:102,400) or (B) trastuzumab (20 to 0.02 μg/ml) and then stained with the appropriate phycoerythrin-conjugated anti-IgG secondary antibody. Mean fluorescence intensity, as a measure of antibody binding to HER2, was plotted. LacZ-VIA as a negative control showed an MFI (mean fluorescence intensity) of less than 50 at all dilutions (1:100 to 1:102,400) (data not shown). [file bcr3204-S1.PDF]

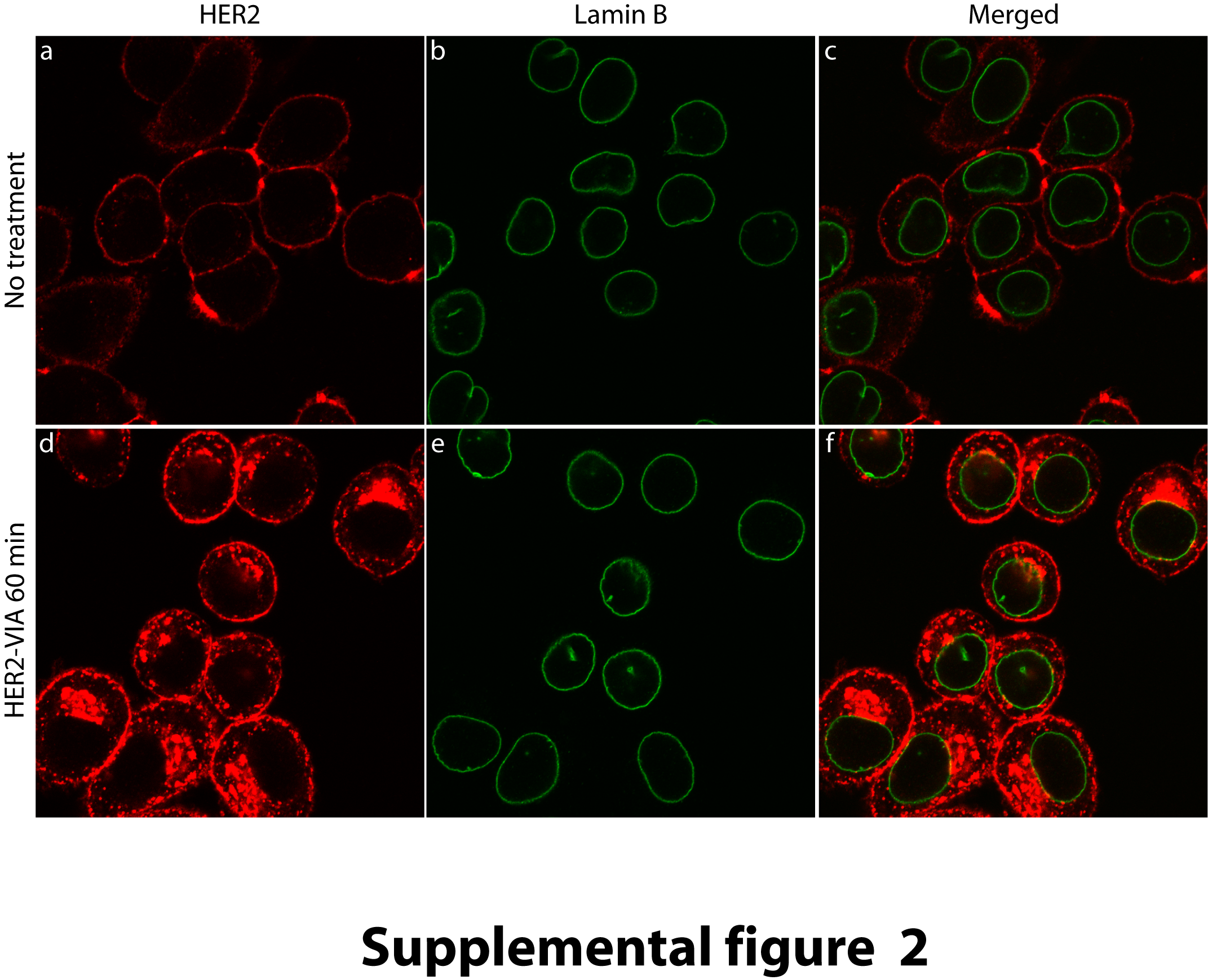

Supplement: Additional file 2 — Figure S2 showing HER2-VIA drives HER internalization to the cytoplasm but not to the nucleus. Confocal images of SK-BR-3 cells were (a to c) left untreated, or (d to f) treated with HER2-VIA for 60 minutes. Cells were stained with anti-HER2 antibody (a, d) and lamin B (b, e). (c, f) Merged pictures. [file bcr3204-S2.TIFF]

## Slide 1
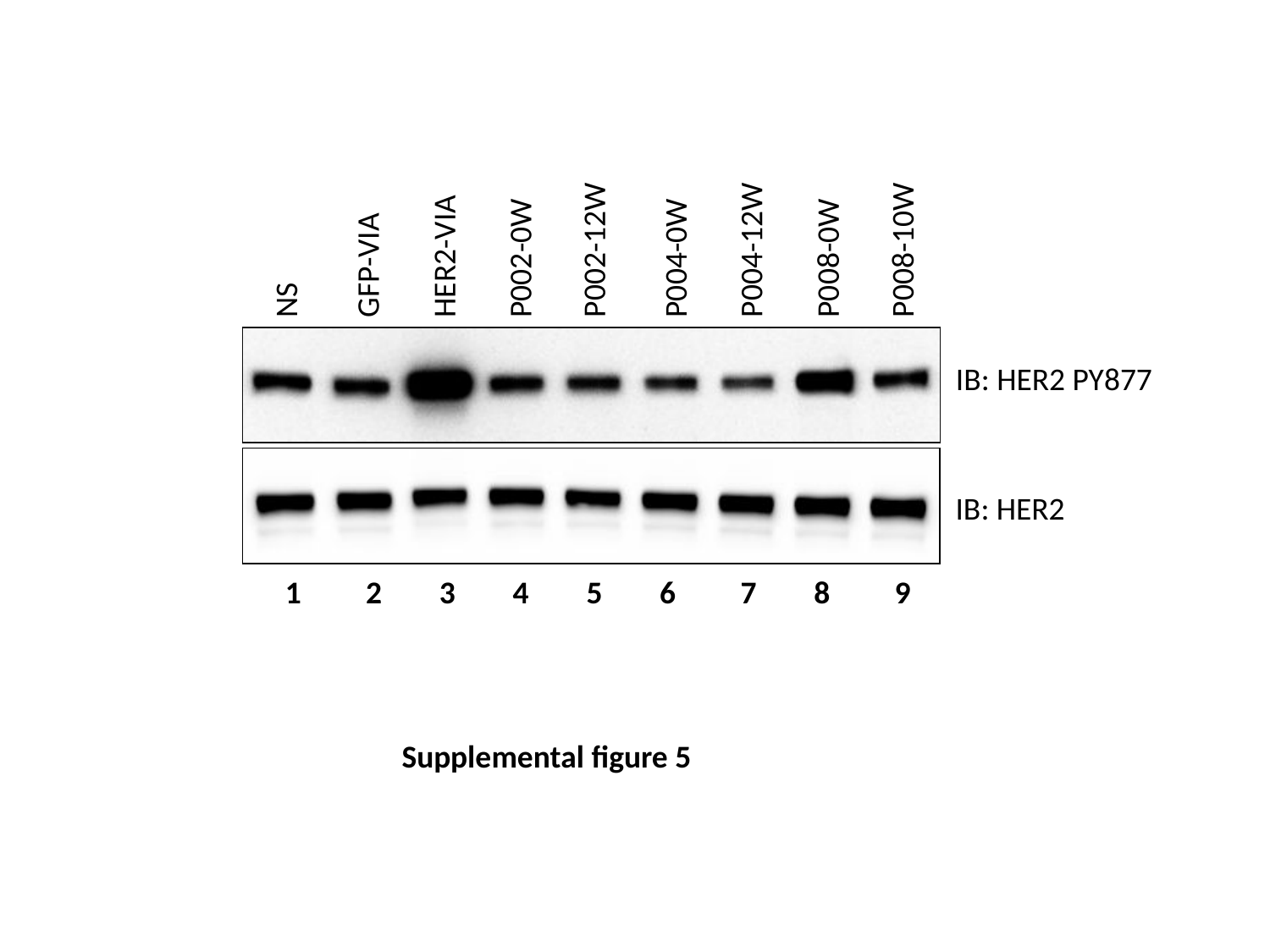

P002-12W
P004-12W
P008-10W
HER2-VIA
P002-0W
P004-0W
P008-0W
GFP-VIA
NS
IB: HER2 PY877
IB: HER2
1 2 3 4 5 6 7 8 9
Supplemental figure 5

Supplement: Additional file 5 — Figure S5 showing the effect of human HER2-specific antibodies on HER2 tyrosine 877 phosphorylation. SK-BR-3 cells were stimulated for 1 hour with the indicated sera from either patients or mice. Protein samples were immunoblotted with anti-HER2 PY877 antibodies (upper panel) or anti-HER2 antibodies (lower panel). [file bcr3204-S5.PPT]
