# Supplementary material for: Polyclonal HER2-specific antibodies induced by vaccination mediate receptor internalization and degradation in tumor cells
Source: Breast Cancer Res. 2012 Jun 7;14(3):R89. doi: 10.1186/bcr3204 (PMC3446352; doi:10.1186/bcr3204)
Supplement: Additional file 3 — Figure S3 showing sucrose inhibits HER2-VIA-induced internalization of HER2. SK-BR-3 cells were treated with 20 µl HER2-VIA and then incubated with FITC 488-conjugated goat anti-mouse antibody on ice. The cells were then exposed to the following conditions and were then imaged by confocal microscopy: (a) incubation on ice for 1 hour; (b) incubation at 37°C for 1 hour; (c) incubation in 0.45 M sucrose on ice; (d) incubation in 0.45 M sucrose at 37°C for 1 hour; (e) incubation in 0.45 M sucrose on ice for 30 minutes followed by washing and then incubation of the cells at 37°C for 1 hour. [file bcr3204-S3.PPT]

## Slide 1
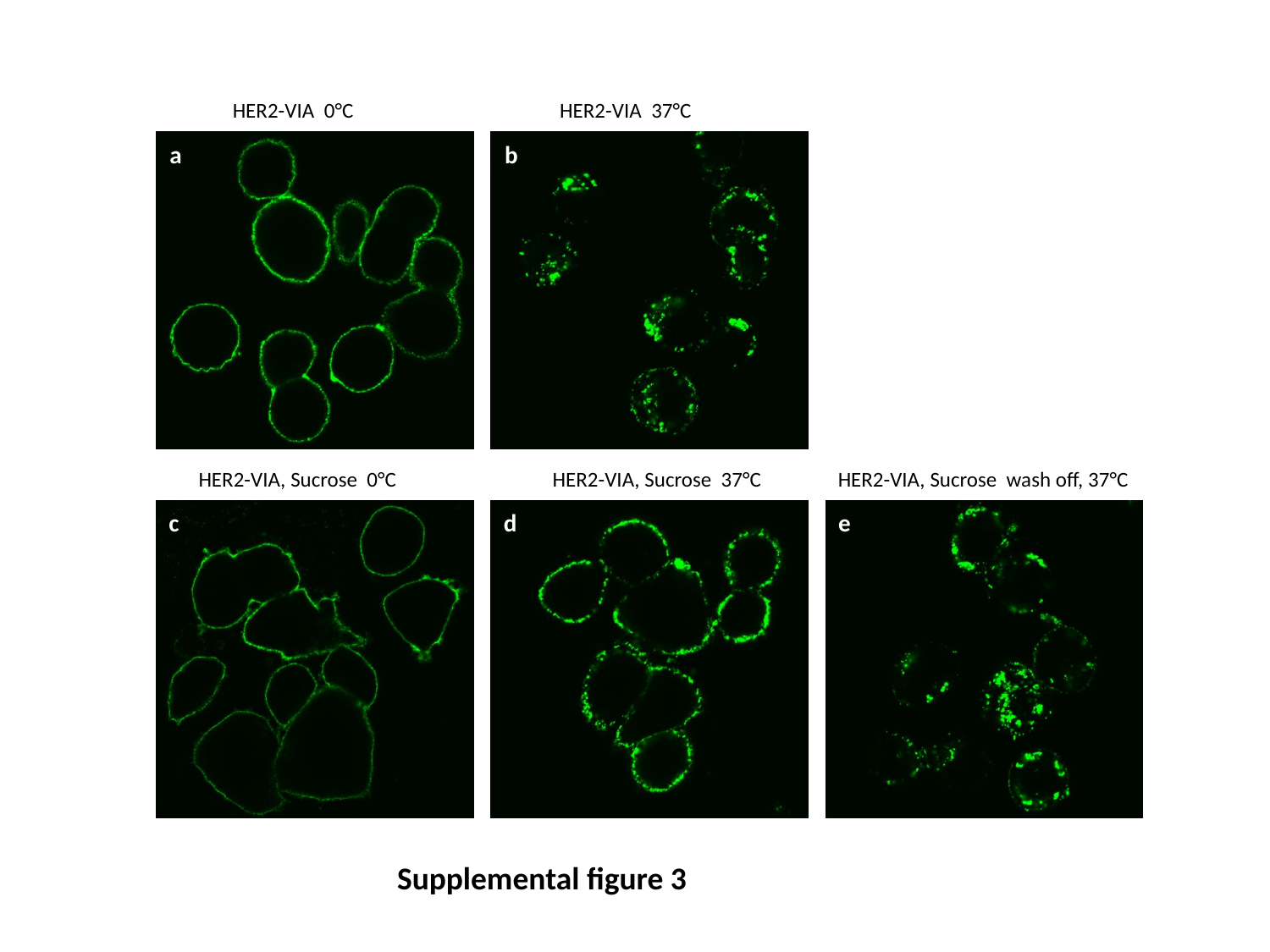

HER2-VIA 0°C
HER2-VIA 37°C
a
b
HER2-VIA, Sucrose wash off, 37°C
HER2-VIA, Sucrose 0°C
HER2-VIA, Sucrose 37°C
c
d
e
Supplemental figure 3
