# Supplementary material for: Polyclonal HER2-specific antibodies induced by vaccination mediate receptor internalization and degradation in tumor cells
Source: Breast Cancer Res. 2012 Jun 7;14(3):R89. doi: 10.1186/bcr3204 (PMC3446352; doi:10.1186/bcr3204)
Supplement: Additional file 4 — Figure S4 showing inhibition of HER2-VIA-induced HER2 ubiquitination by lapatinib. SK-BR-3 cells were pretreated with the proteasome inhibitor MG132 (10 µM) and lapatinib for 30 minutes before HER2-VIA application for 2 hours. After the indicated treatment, cells were lysed and HER2 was precipitated using anti-HER2 rabbit antibody 29D8. Precipitated proteins were subjected to western blot analysis. Upper panel: ubiquitinated HER2; lower panel: total HER2 visualized by anti-HER2 rabbit antibody 29D8. [file bcr3204-S4.PPT]

## Slide 1
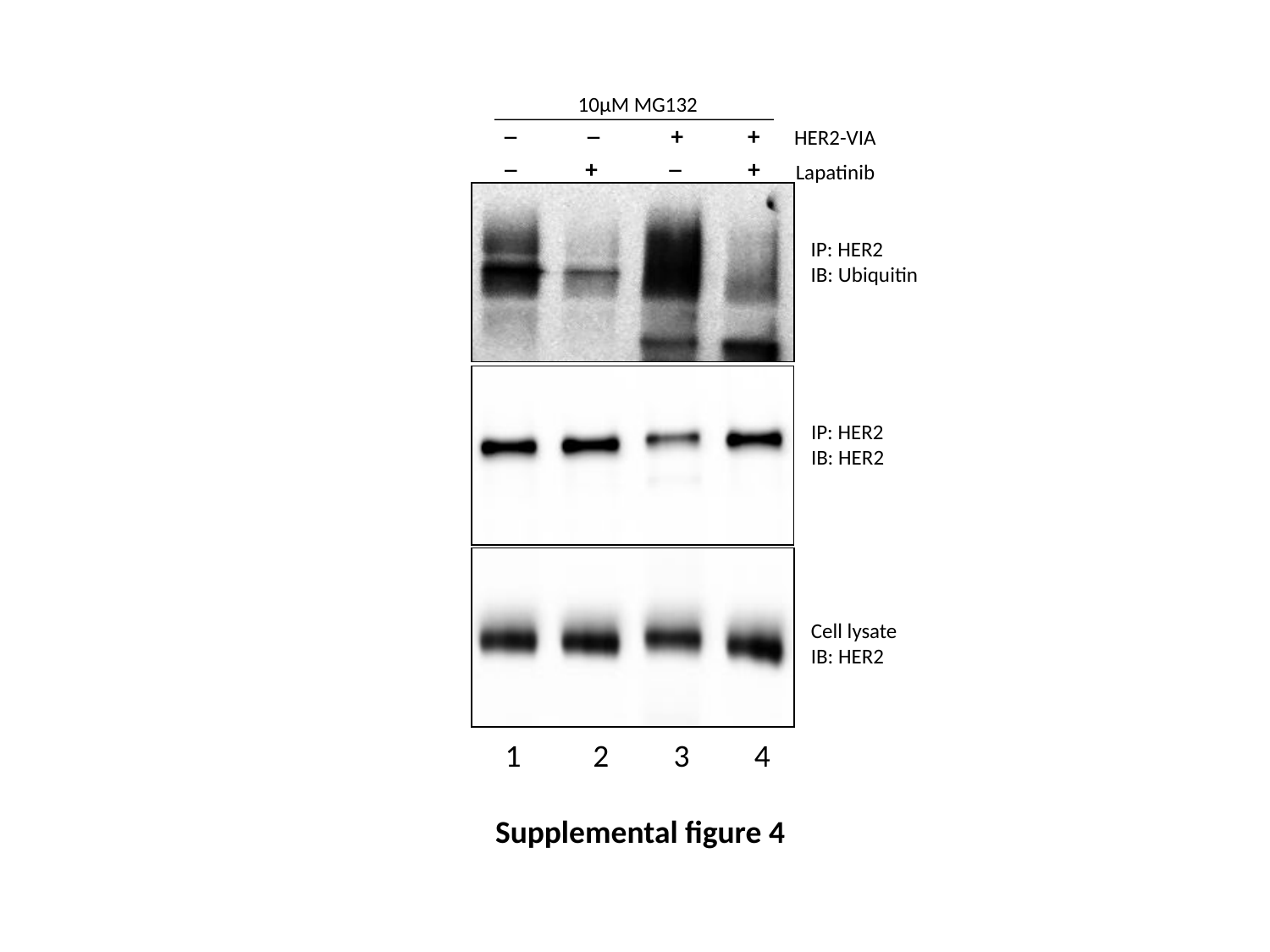

10µM MG132
_
_
+
+
HER2-VIA
_
_
+
+
Lapatinib
IP: HER2
IB: Ubiquitin
IP: HER2
IB: HER2
Cell lysate
IB: HER2
1 2 3 4
Supplemental figure 4
